# The casual relationship between autoimmune diseases and multiple myeloma: a Mendelian randomization study

**DOI:** 10.1007/s10238-024-01327-x

**Published:** 2024-04-02

**Authors:** Peipei Jin, Xiaoqing Jin, Li He, Wen Liu, Zhuo Zhan

**Affiliations:** 1 Second School of Clinical Medicine, Zhongnan Hospital of Wuhan University, Wuhan, 430071 Hubei China; 2 Emergency Center, Zhongnan Hospital of Wuhan University, Wuhan, 430071 Hubei China; 3 Department of Hematology, Zhongnan Hospital of Wuhan University, 169 Donghu Road, Wuhan, 430071 Hubei China

**Keywords:** Mendelian randomization study, Genome-wide association study, Multiple myeloma, Autoimmune diseases

## Abstract

Observational studies showed possible associations between systemic lupus erythematosus and multiple myeloma. However, whether there is a casual relationship between different types of autoimmune diseases (type 1 diabetes mellitus, rheumatoid arthritis, systemic lupus erythematosus, psoriasis, multiple sclerosis, primary sclerosing cholangitis, primary biliary cirrhosis, and juvenile idiopathic arthritis) and multiple myeloma (MM) is not well known. We performed a two-sample Mendelian randomization (MR) study to estimate the casual relationship. Summary-level data of autoimmune diseases were gained from published genome-wide association studies while data of MM was obtained from UKBiobank. The Inverse-Variance Weighted (IVW) method was used as the primary analysis method to interpret the study results, with MR-Egger and weighted median as complementary methods of analysis. There is causal relationship between primary sclerosing cholangitis [OR = 1.00015, 95% CI 1.000048–1.000254, *P* = 0.004] and MM. Nevertheless, no similar causal relationship was found between the remaining seven autoimmune diseases and MM. Considering the important role of age at recruitment and body mass index (BMI) in MM, we excluded these relevant instrument variables, and similar results were obtained. The accuracy and robustness of these findings were confirmed by sensitivity tests. Overall, MR analysis suggests that genetic liability to primary sclerosing cholangitis could be causally related to the increasing risk of MM. This finding may serve as a guide for clinical attention to patients with autoimmune diseases and their early screening for MM.

**Supplementary Information:**

The online version contains supplementary material available at 10.1007/s10238-024-01327-x.

## Introduction

Multiple myeloma (MM) is the second most common Hematological malignancy only after leukemia [1]. It is a malignant proliferative disease, characterized by an uncontrolled increase in plasma cell proliferation in the bone marrow, accompanied by a large production of monoclonal immunoglobulins, leading to damage to multiple organs or tissues (bone lesions, kidney injury, anemia, and hypercalcemia etc.) [2]. Due to population growth and aging, the incidence and mortality of MM are increasing rapidly, with approximately 588,000 patients diagnosed worldwide each year [3]. As is well recognized, most people diagnosed with MM in the general population are over 65 years old or physically inactive [4], leading to a heavy disease burden. The prognosis for MM patients is less favorable with a median survival of 3–4 years, and even though treatment options for MM are improving, MM remains incurable [2]. At present, the viewpoint that cytogenetic abnormalities contribute to MM pathogenesis and progression is generally accepted [5]. Based on this fact, the causal relationship between other possible risk factors and MM remains to be unveiled.

Recently, there has been growing interest in investigating the relationship between autoimmune diseases and MM. A total of 15 cases of SLE (systemic lupus erythematosus) comorbid with MM have been reported cumulatively in PUBMED and Mendeley from 2000 to 2023, [6] with MM occurring after SLE in most cases. The median age of diagnosis of them was 50 years (10 years earlier than the age of conventional diagnosis of MM), which in a sense suggested the promotion of SLE on the occurrence of MM. What’s more, meta-analyses have demonstrated that certain autoimmune diseases may increase the risk of developing MM [7]. According to a meta-analysis of case–control and cohort studies in 2014, rheumatoid arthritis does not appear to modify the risk of myeloma, while Pernicious anemia and ankylosing spondylitis might be the underlying risk factors for the development of myeloma [8]. However, conflicting results have been reported. For example, a large population-based study in 2021 found that a history of autoimmune disease was associated with a reduced risk of progression from MGUS to MM [9]. Therefore, we want to reveal the causal relationship between autoimmune diseases and MM from another perspective. Unfortunately, there is a lack of large randomized controlled trials (RCT) revealing the causal relationship between autoimmune diseases and MM.

Randomized Controlled Trials (RCT) are the gold standard for generating clinical casual evidence, yet in reality, there still exist some limitations such as implementation difficulties, ethical constraints, etc. [10] As another classic clinical research method, observational research usually has a bias, and is less reliable due to potential confounding factors and reverse causality [11]. At this point, Mendelian randomization (MR) study begins to emerge, bridging the gap between conventional research methods. The principle of random allocation of alleles in a Mendelian randomized study is similar to the randomized grouping method in RCT studies [12]. And MR can use single nucleotide polymorphisms (SNP) as a proxy for exposure to evaluate the cause effects between the exposures and outcomes of interest free from confounding factors [13]. Additionally, MR has several advantages over traditional observational studies. Firstly, it can avoid reverse causation because genetic variants are determined at birth and cannot be affected by disease status later in life. Secondly, MR can investigate the long-term effects of an exposure on an outcome. Unlike observational studies, which typically have short follow-up periods, MR can study outcomes over a longer time period. Thirdly, MR can investigate causal relationships between exposures and diseases that cannot be directly manipulated due to ethical concerns or practical difficulties. For these advantages, MR has been increasingly used in various fields, including cancer, cardiovascular diseases, and mental disorders. Furthermore, MR has provided valuable causal evidence that has helped guide the development of new interventions for the prevention and treatment of diseases.

In this study, it aims to explore the causal relationship between autoimmune diseases and MM by using a two-sample MR study, and to identify specific risk groups early in order to enable early intervention and prevention.

## Methods

### Study design

MR is an epidemiological approach that aims to deduce causal relationships by using genetic variants, specifically single-nucleotide polymorphisms (SNPs), as instrumental variables for exposure [14]. The three assumptions on which genes selection relies include the relevance hypothesis, the assumption of independence, and the exclusivity restrictions [15]: ①SNPs selected as a genetic instrument should be robustly associated with the exposure ②As a bridge, SNPs should be independent of any possible confoundings ③ SNPs cannot be directly linked to outcome, and can only affect the outcome through exposure. In the present study, a two-sample Mendelian randomization analysis was conducted to investigate certain causal relationships between the eight included autoimmune diseases and MM. For this purpose, SNPs that were strongly associated with each autoimmune disease were selected as instrumental variables.

### Data resources

*Autoimmune diseases data source:* The data for the eight autoimmune diseases included in the study (namely type 1 diabetes mellitus, rheumatoid arthritis, systemic lupus erythematosus, psoriasis, multiple sclerosis, primary sclerosing cholangitis, primary biliary cirrhosis, and juvenile idiopathic arthritis) were obtained from the largest relevant publicly available genome-wide association study (GWAS) in summary-level format.[16–22] Nevertheless, as for JIA, the information of genetic variants was obtained from published studies[16]. The summary data on genetic variants about a variety of exposure traits can be downloaded from IEU OpenGWAS project website. Detailed study information for each variant such as year, author, sample size, population, consortium, etc. could be found on https://gwas.mrcieu.ac.uk/ [23]. The GWAS-ID for each are listed below: “ebi-a-GCST005536” for type 1 diabetes mellitus, “bbj-a-74” for rheumatoid arthritis, “ebi-a-GCST003156” for systemic lupus erythematosus, “ebi-a-GCST005527” for psoriasis, “ieu-b-18” for “multiple sclerosis”, “ieu-a-1112” for “primary sclerosing cholangitis”, and “ebi-a-GCST005581” for primary biliary cirrhosis. Further details of the GWASs are listed in the Table 1.

**Table 1:** The details of genome-wide association studies used to obtain the genetic variants for each autoimmune disease examined

Autoimmune disease	GWAS ID	snps	Case	Control	Total snps	Sample size	Snps in outcome
Type 1 diabetes mellitus	ebi-a-GCST005536	38	6,683	12,173	138,229	18,856	37
Rheumatoid arthritis	bbj-a-74	86	3,636	15,554	9,739,303	19,190	73
Systemic lupus erythematosus	ebi-a-GCST003156	58	7,219	15,991	7,071,163	23,210	54
Psoriasis	ebi-a-GCST005527	90	10,588	22,806	138,661	33,394	75
Multiple sclerosis	ieu-b-18	94	47,429	68,374	6,304,359	115,803	93
Primary sclerosing cholangitis	ieu-a-1112	29	2,871	12,019	7,891,603	14,890	24
Primary biliary cirrhosis	ebi-a-GCST005581	28	2,861	8,514	119,756	11,375	27
Juvenile idiopathic arthritis	33,106,285	21	2751	15,886	7,500,000	18,637	21
Multiple myeloma	ieu-b-4957	NA	601	372,016	8,615,746	372,617	NA

*MM data source:* The summary-level data on MM was gained from UKbiobank, a recent GWAS of 372,617 European individuals with 601 cases and 372,016 controls[23]. Furthermore, the detailed information of each SNPs is available for download and analysis. Since autoimmune disease and MM are from different consortia, there is no sample overlap. The specific information about the trait is also listed in the Table 1.

### Selection of genetic instrumental variables

Firstly, to verify the relevance hypothesis, SNPs that were strongly (*p* < 5 × 10^–8)^ and independently (r2 < 0.01) associated with each autoimmune disease were selected to further remove the linkage disequilibrium by setting Clump = TRUE and r2 < 0.01, kb = 10,000. After that, we excluded the weak instrumental variables by calculating the degree of explanation [*R*2 = 2*eaf*(1-eaf)*beta2] and the F-statistic [beta2/se2] reflecting the instrumental strength for each SNP–exposure association [24, 25]. Eaf stands for the effect allele frequency (eaf) of the SNP, beta is the estimated effect of SNP on trait, and se represents the standard error. SNPs with F statistic less than 10 is defined as a weak instrumental variable. Second, we confirmed the assumption of independence by investigating whether the selected SNPs were correlated with confounding factors such as age at recruitment and BMI, which have been discovered as risk factors for MM. The associations of these SNPs with BMI were obtained from a meta-analysis of GWASs [26]. MM is known to have a higher incidence in older populations. However, this group also faces a competing risk of both mortality and MM, which may lead to a survival bias in studies. This bias should be taken into consideration while analyzing the data to ensure accurate results. [27] Due to this bias, we excluded the SNPs associated with survival which were proxied by age at recruitment from the UK Biobank GWAS (http://www.nealelab.is/uk-biobank) as described before [24]. The associations with age at recruitment to the UK Biobank (http://www.nealelab.is/uk-biobank) were adjusted for the first 20 principal components, age, age2, sex, age × sex and age2 × sex. Given the number of SNPs that were predictive for autoimmune diseases and MM, we applied a Bonferroni correction (0.05/number of SNPs) to assess the relevance of BMI or age at recruitment. Subsequently, we selected more than 20 SNPs for each exposure factor, which were deemed suitable for further MR analysis.

### MR analysis and sensitivity analysis

Information on SNPs, effect allele, other alleles, effect sizes, se, *P* values and eaf are required for MR. After extracting the other required parameters from the outcome data matching the SNPs of the instrumental variables, rigorous data harmonization was performed to ensure that the effects of the SNPs on exposure and outcome corresponded to the same alleles.

The inverse-variance weighting (IVW) method was used as the major analysis method to interpret the study results. Since it is difficult to verify the ‘exclusion–restriction’ assumption, MR-Egger, weighted median and Mendelian Randomization Pleiotropy RESidual Sum and Outlier (MR-PRESSO) was employed as auxiliary verification. In this study, as eight autoimmune diseases were studied, the significance level was set at 0.00625 (0.05/8) to correct for multiple comparisons. Results with *P* values below this threshold were considered as strong evidence of associations, and results with *P* values between 0.00625 and 0.05 were regarded as suggestive associations. Results that were significant before but not after correction for multiple comparison were also considered as suggestive associations.

To further monitor or eliminate bias in the study, sensitivity analysis was performed, which includes tests for heterogeneity, Mendelian Randomization Pleiotropy RESidual Sum and Outlier (MR-PRESSO) and leave-one-out analysis. MR-PRESSO performed a global test to detect horizontal pleiotropy, and if necessary, could correct for potential pleiotropic outliers via outliers removal. However, it is imperative to underscore that while statistical pleiotropy can be corrected, biological pleiotropy cannot be eliminated. The Leave-one-out analysis progressively eliminates each SNP, calculates the effect of the remaining SNPs, and observes the change in the results. The MR analysis was accomplished by TwoSampleMR package in R (Version4.2.1). No ethical approval is required since the data we used is publicly available summary data.

## Results

### IVs selection

This study included 38 SNPs for type 1 diabetes, 86 SNPs for rheumatoid arthritis, 58 SNPs for Systemic lupus erythematosus, 90 SNPs for Psoriasis, 94 SNPs for multiple sclerosis, 29 SNPs for primary sclerosing cholangitis, 28 SNPs for primary biliary cirrhosis and 21 SNPs for juvenile idiopathic arthritis, as they were strongly and independently associated with the respective autoimmune diseases. The F-statistics of these SNPs ranged from 30 to 375, which indicates that the bias caused by weak instruments is likely negligible.

Some SNPs were not available for the outcome, and no proxy SNPs were available for analysis as we had a sufficient number of genetic tools. Table 1 provides information on the number of eligible SNPs remaining after merging the SNPs for both exposure and outcome.

After extracting instrumental variables and merging them with GWAS data of MM, we performed data harmonization to ensure that the direction of exposure and outcome was consistent for the MR analysis. When assessing survival, all SNPs were included in the analysis as no associations with age at recruitment were identified (*P* < 0.05/number of SNPs). Regarding the confounding factor BMI, we removed two SNPs (rs1701704, rs3184504) that predicted Type 1 diabetes and one SNP (rs4766578) that predicted juvenile idiopathic arthritis which were found to be associated with BMI (Bonferroni correction p-value 0.05/37and 0.05/21). The harmonized data and *P* value of age at recruitment could be obtained in supplementary Table 1. As shown in supplementary Table 2 which is accessible in the supplementary materials, the results were similar before and after the removal of these SNPs.

### Effects of eight autoimmune diseases on MM

**Fig. 1:**
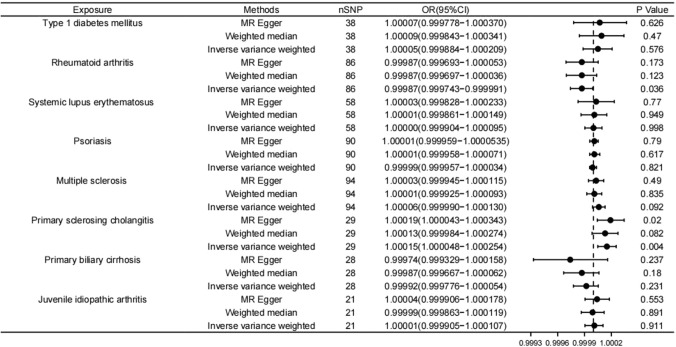
Forest plot of Mendelian randomization (MR) estimates for 8 autoimmune diseases with MM. The causal effect between each autoimmune disease and MM was estimated using three different MR methods. Odd ratio (OR) with 95% confidence intervals indicates the odds ratio of having MM in patients with autoimmune disease compared to those without them

**Fig. 2:**
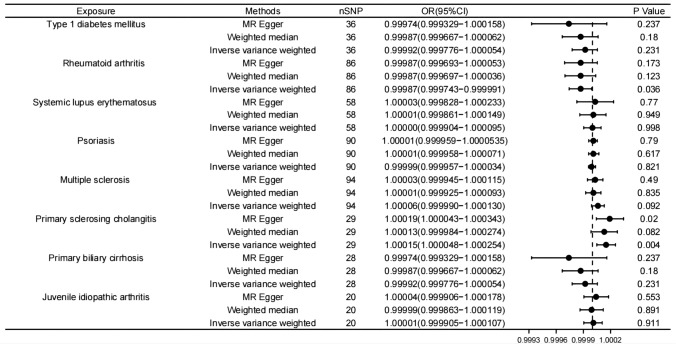
Forest plot of Mendelian randomization (MR) estimates for 8 autoimmune diseases with MM after adjusting for BMI confounders. The causal effect between each autoimmune disease and MM was estimated using three different MR methods. Odd ratio (OR) with 95% confidence intervals indicates the odds ratio of having MM in patients with autoimmune disease compared to those without them

According to the primary results of IVW, there was a statistically significant correlation between an elevated risk of MM and an increased risk of primary sclerosing cholangitis (IVW *p* value = 0.004) whose OR was 1.000151 (95%CI 1.000048–1.000254). Type 1 diabetes, Systemic lupus erythematosus, Psoriasis, Multiple sclerosis, Primary sclerosing cholangitis, and Juvenile idiopathic arthritis showed positive correlations with MM, but none of them reached statistical significance. A negative correlation was observed between rheumatoid arthritis and MM before correction (IVW *p* value = 0.036), with an odds ratio (OR) of 0.999867 (95%CI 0.999744–0.999991). However, the result lost statistical significance after Bonferroni correction, which may remind a suggestive relationship. Similarly, a negative correlation was observed between primary billary cirrhosis and MM, but it did not reach statistical significance (IVW *p* value = 0.231). Theses results are displayed in Figs. 1 and 2.

### Sensitivity analyses for MR analysis

**Fig. 3:**
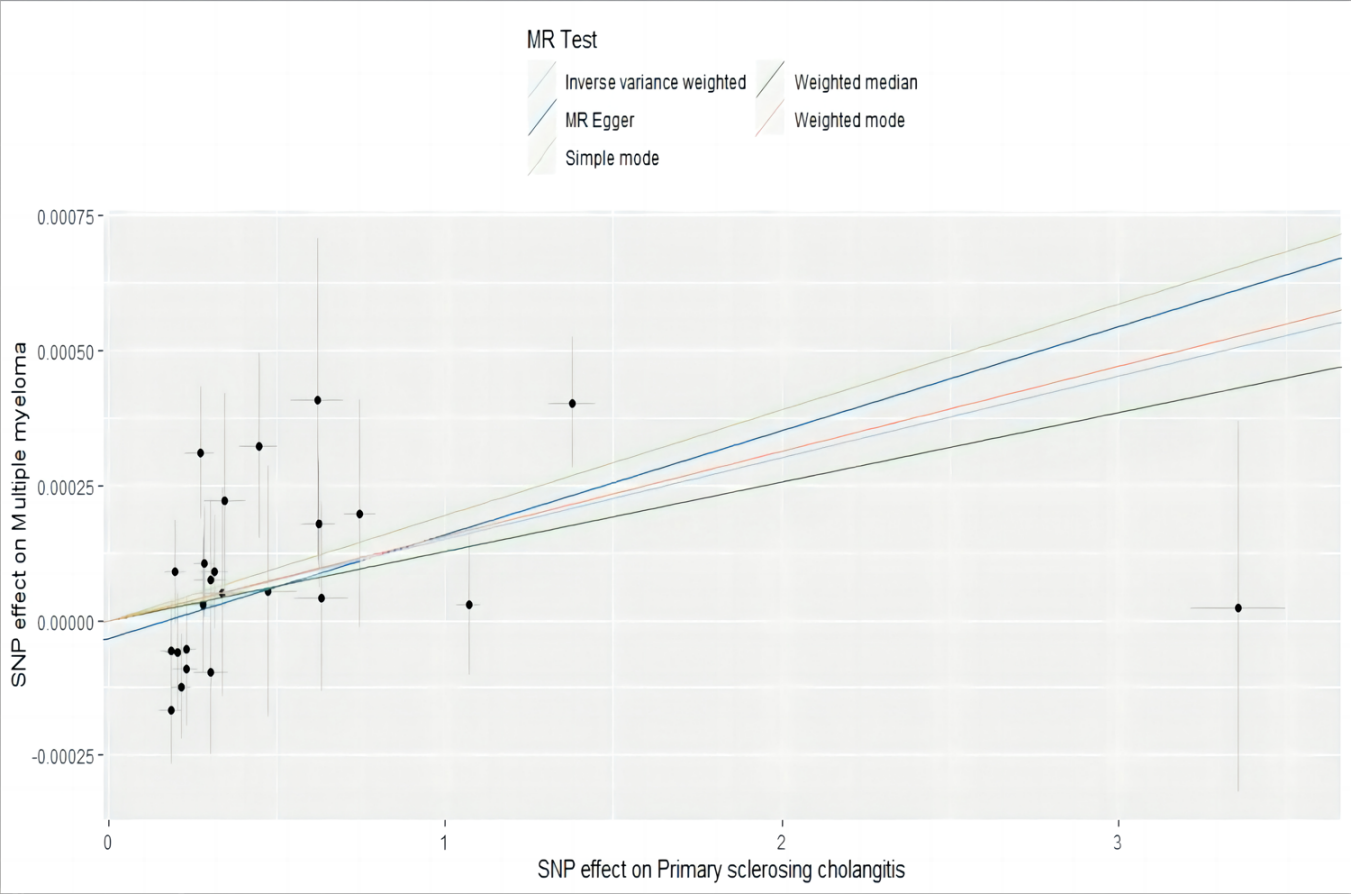
Scatter plot of SNP effect on primary sclerosing cholangitis and MM. Each point on the graph represents an SNP site. The abscissa is the effect of SNP on exposure, the ordinate is the effect of SNP on outcome, and the colored lines represent the MR fitting results

**Fig. 4:**
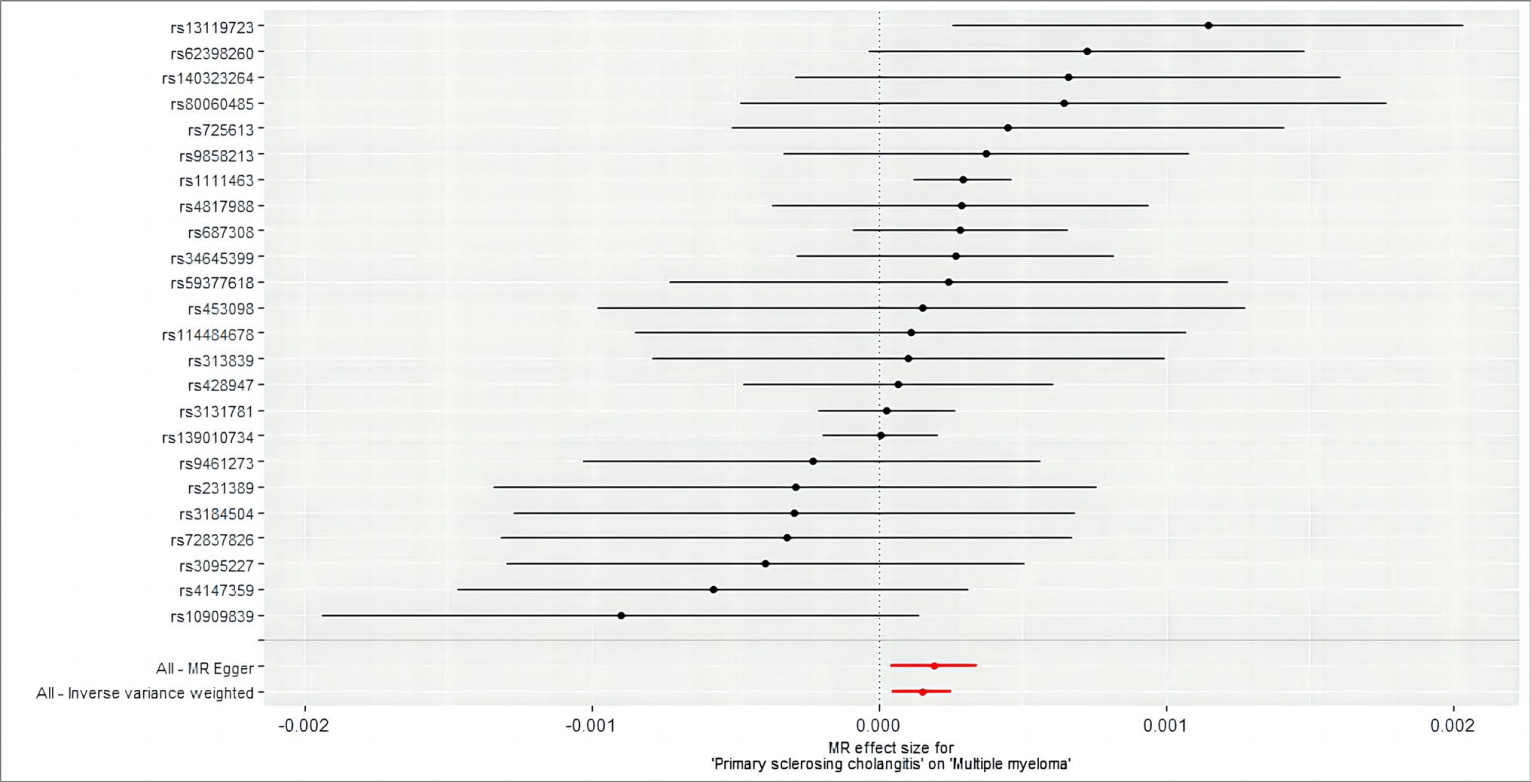
Forest plot of the causal effect of each individual primary sclerosing cholangitis instrument SNP with MM. To the left of the dotted line indicates a possible protective factor for MM, while to the right indicates a possible facilitating factor for MM. The solid red line shows the overall effect

**Fig. 5:**
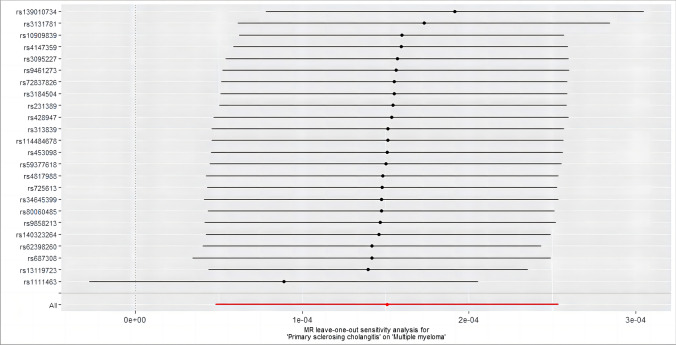
Forest plot of the causal effect on MM outcomes after excluding instrument SNPs for primary sclerosing cholangitis on a case-by-case basis. All scatter points are to the right of the dashed line, indicating that excluding any SNP has no significant effect on the results

SNP-exposure and SNP-outcome were selected as two groups of samples with different populations and sequencing methods, and a heterogeneity test was performed to assess their differences. Sensitivity analysis suggests no heterogeneity or pleiotropy in them (*p* > 0.05). The bias resulting from genetic pleiotropy was evaluated using MR-Egger regression analysis. The intercept of the MR-Egger regression line can estimate the magnitude of directional pleiotropy, which was calculated by mr_pleiotrophy_test. The results showed no evidence of horizontal pleiotropy between primary sclerosing cholangitis and MM, as shown in Fig. 3. After obtaining individual SNP results using mr_singlesnp, a forest plot of the effect of each SNP was created by mr_forest_plot. Figure 4 shows the forest plot of individual SNP results for primary sclerosing cholangitis. However, it should be noted that the statistical pleiotropy cannot exclude biological pleiotropy. We then used mr_funnel_plot to draw funnel plots and analyzed the heterogeneity based on the results of the individual SNPs obtained before. MR leave − one − out sensitivity analysis for them was adopted to gain Cross-validation. (Fig. 5 shows the leave − one − out plot of psc). The essence of this analysis is similar to the statistical iterative method of screening predictor variables when constructing a predictive model, whereby individual SNPs were systematically removed to see if they have a significant effect on the results. Detailed visualization results of the other seven autoimmune diseases can be acquired in the supplementary materials.

## Discussion

The two-sample MR analysis in the present study first examined the potential correlation between autoimmune diseases and MM. Recently, an MR study of genetic predisposition to autoimmune diseases and COVID-19 was published [16], providing reference for our research. After that, genetic evidence was provided that genetically predicted primary sclerosing cholangitis was associated with MM, but no evidence showed that genetic liability to the remaining seven autoimmune diseases was related to MM. Primary sclerosing cholangitis might be associated with a higher risk of developing MM, as indicated by our MR analysis.

Several prior observational studies or meta-analyses have provided the basis for our research by regarding the causal relationship between autoimmune disease and MM [7–9, 28]. One example of such prior studies is a 2012 publication which reported an elevated risk of myeloma after certain autoimmune diseases, [29] and a meta-analysis indicated that SLE was correlated with increased risk of MM [28, 30]. While observational studies have limitations, these findings offer preliminary evidence, supporting further high-quality studies to ascertain causality. In reality, there are some mechanisms that may explain the correlation between them [31, 32]. Firstly, the role of immune system in the development of MM has been a subject of interest to researchers. Interleukin-6 (IL-6), a multifunctional cytokine, plays an important role in immune and inflammatory responses [33, 34]. However, the persistent production of its dysregulation can lead to the development of various autoimmune and chronic inflammatory diseases. IL-6 is also a crucial growth factor in myeloma cells, which is indispensable in MM tumorigenesis, maintenance of malignant cell clones, and monoclonal immunoglobulin production [35]. Thus, we propose that autoimmune diseases and MM are correlated. Secondly, autoimmune diseases are characterized by the body's immune response to its own antigens [36].The immune system becomes activated when a pathogen invades the body, and the presence of antigens similar to the pathogen in the body triggers an autoimmune response, which in turn activates B cells [37]. Excessive B-cell activity facilitates the escape of abnormal B-cell clones from the normal regulatory system. Meanwhile, the hallmark of MM is the uncontrolled proliferation of plasma cells in the bone marrow [3]. As such, the above traits suggest a possible association between autoimmune disease and the development of MM. Nevertheless, whether there is an exact causal relationship between them remains elusive.

The conclusion draw in our study regarding the causal relationship between primary sclerosing cholangitis and MM can also be explained by the mechanism described above. For example, autoantibodies in the serum of PSC patients can bind to the antigens of bile duct epithelial cells, stimulating the related signal transduction and receptor expression, and ultimately releasing various inflammatory factors, chemokines and growth factors from bile duct epithelial cells [38, 39]. Furthermore, a search of the relevant literature revealed that in 2006 Vincenzo Fontana reported the first case of primary sclerosing cholangitis (PSC) complicated by plasmacytosis (PCD), which was initially stable for 3 years but progressed to be consistent with MM, and the MM went into clinical remission after liver transplantation [40]. This case may suggest that chronic cirrhosis of the liver antigenic stimulation contributed to monoclonal gammopathy of undetermined significance (MGUS) and subsequent conversion to MM. Since primary sclerosing cholangitis complicating plasma cell dyscrasia (PCD) is often associated with other chronic liver diseases, patients with various chronic liver diseases complicated by PCD may obtain similar benefits. [40] Therefore, our study provides evidence supporting the hypothesis that there exists a causal relationship between primary sclerosing cholangitis and MM.

Despite the fact that MR designs are less susceptible to confounding factors than observational studies [11], some limitations still need to be considered. Firstly, due to the lack of GWAS data, only a limited number of autoimmune diseases were included in this study, which may limit the accuracy of drawing conclusions. Secondly, even though we selected genetic instruments strongly predicted the exposures supported by high F-statistics there is still potential for our hypothesis of relevance and exclusion restriction not being entirely tested. Thirdy, even after adjusting for possible confounders, there may still be some unobserved bias. However, most SNPs used were not related to potential confounders, such as BMI, nor had any impact on the outcome via other pathways, such as age at recruitment. Similarly, we cannot avoid the existence of biological pleiotropy even if we have verified the statistical horizontal pleiotropy. Lastly, the GWAS used in this study mainly concerns individuals of European descent. Therefore, we should be cautious when applying these findings to other populations.

## Conclusion

In conclusion, our MR study provides evidence supporting a possible causal relationship between primary sclerosing cholangitis and the development of MM from a genetic perspective. Although the effect size of the observed association was modest, our findings offer valuable insights into the pathogenesis of MM. Conducting further analyses in a larger MM cohort will help obtain more accurate evidence regarding those correlations. A better clinical focus on follow-up of patients with autoimmune diseases would facilitate early screening and management of MM.

## Supplementary Information

Below is the link to the electronic supplementary material.

Supplementary file1 (DOCX 878 kb)

Supplementary file2 (XLSX 133 kb)

Supplementary file3 (XLSX 23 kb)

## Data Availability

All the data used in this study had been publicly available.The corresponding websites https://gwas.mrcieu.ac.uk/ were listed in the manuscripts.
