# Supplementary material for: The casual relationship between autoimmune diseases and multiple myeloma: a Mendelian randomization study
Source: Clin Exp Med. 2024 Apr 2;24(1):65. doi: 10.1007/s10238-024-01327-x (PMC10987346; doi:10.1007/s10238-024-01327-x)
Supplement: Supplementary file 1 — Supplementary file1 (DOCX 878 kb) [file 10238_2024_1327_MOESM1_ESM.docx]

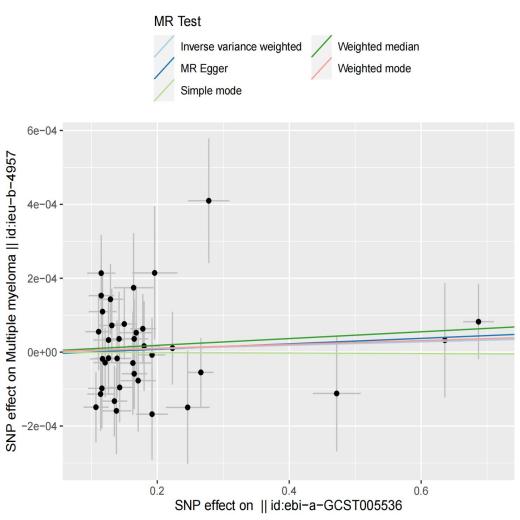

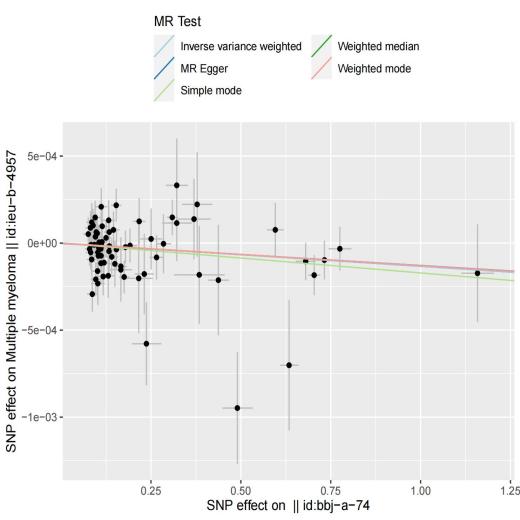


A B


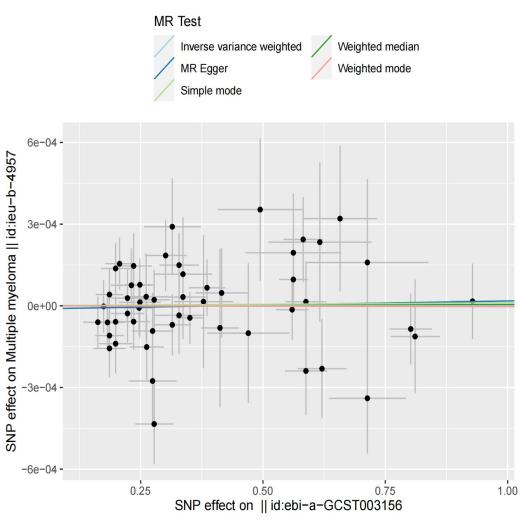

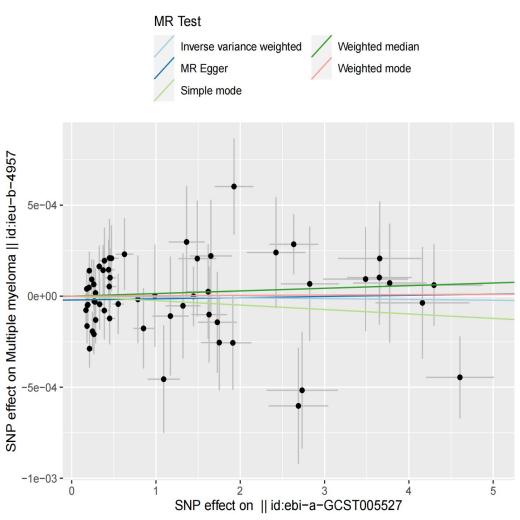


C D


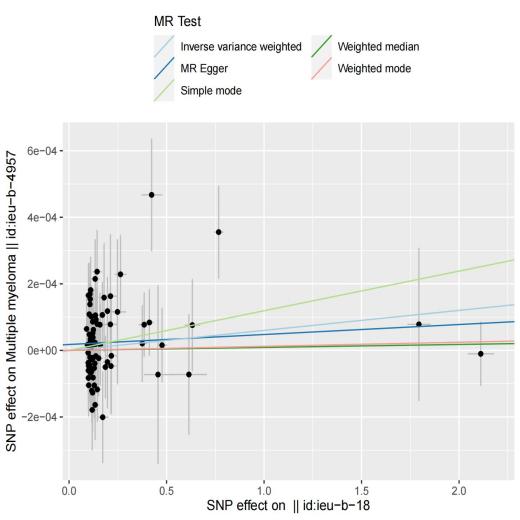

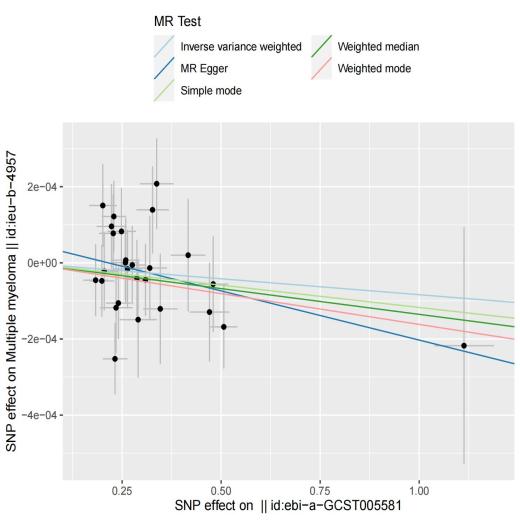


E F


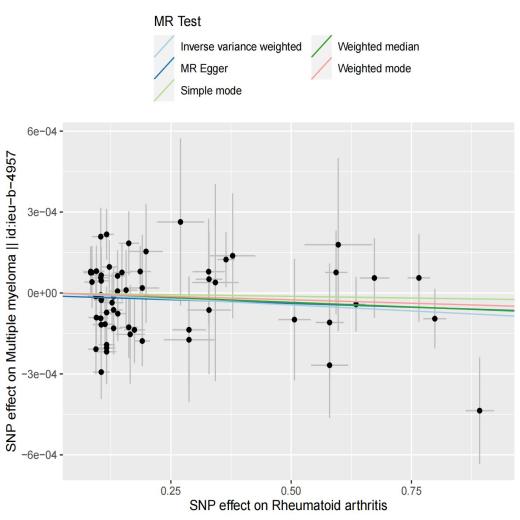


G

**Supplementary figure1**. Scatter plots of causality on MM. The slope of each line corresponding to the estimated MR effect in different models. (A)Type 1 diabetes mellitus;(B)Rheumatoid arthritis;(C)Systemic lupus erythematosus ;(D)Psoriasis;(E)Multiple sclerosis ;(F)Primary biliary cirrhosis;(G)Juvenile idiopathic arthritis.


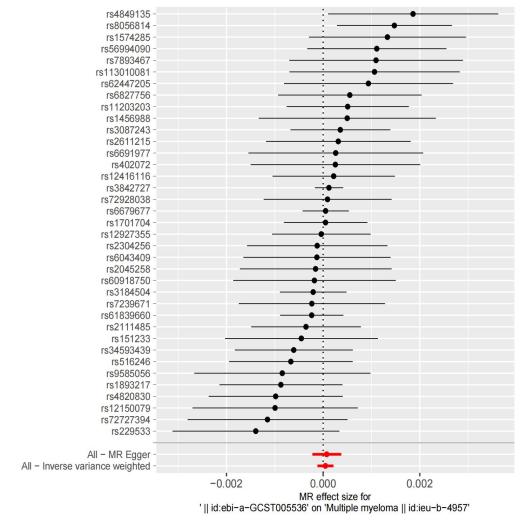

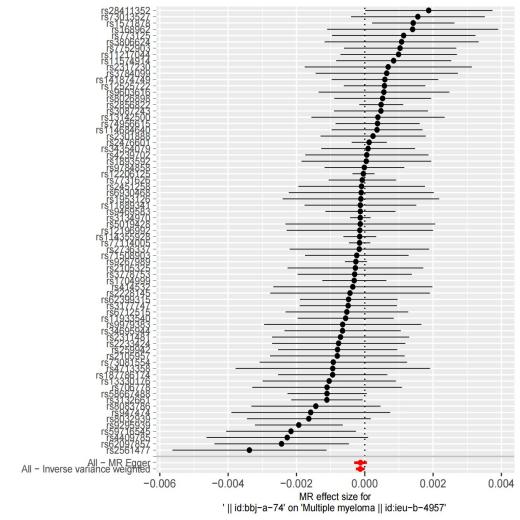


A B


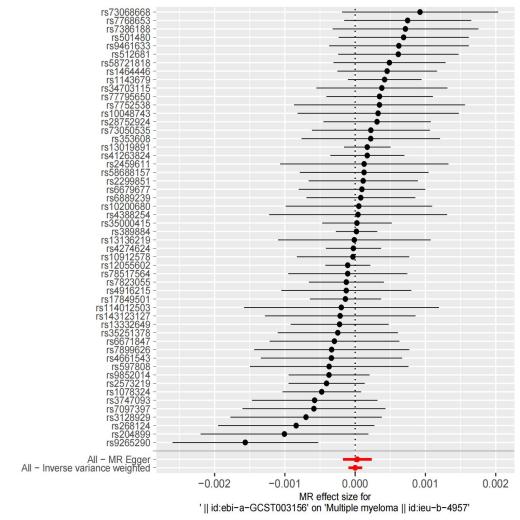

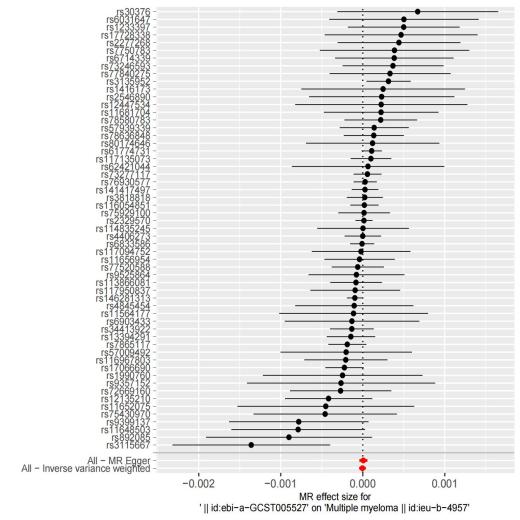


C D


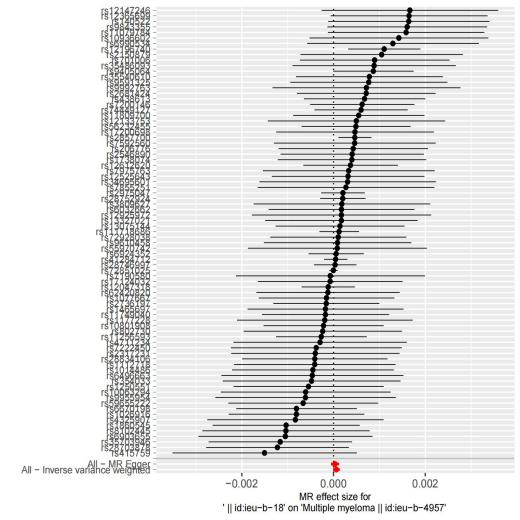

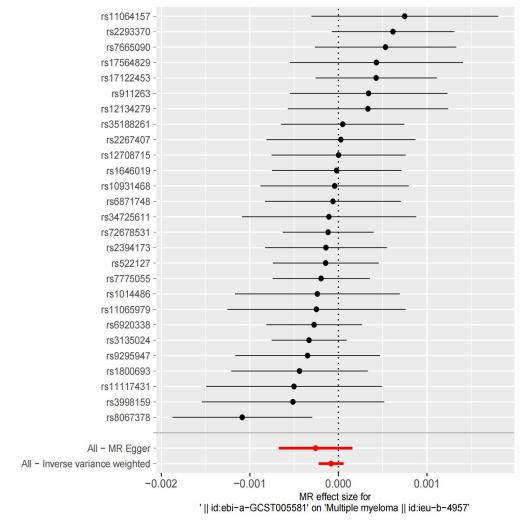


E F


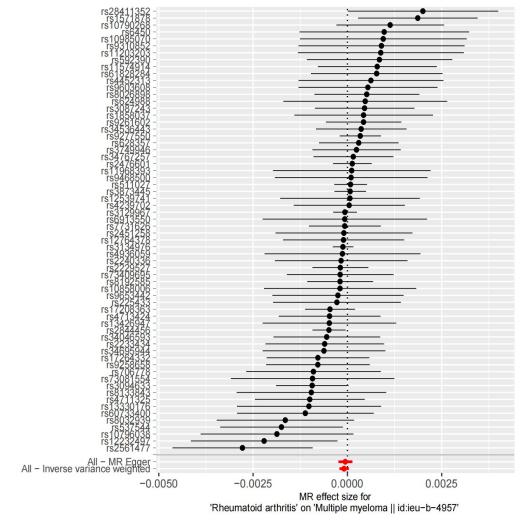


G

**Supplementary figure2**. Forest plot of the causal effect of each individual autoimmune diseases instrument SNP with MM. (A)Type 1 diabetes mellitus;(B)Rheumatoid arthritis;(C)Systemic lupus erythematosus ;(D)Psoriasis;(E)Multiple sclerosis ;(F)Primary biliary cirrhosis;(G)Juvenile idiopathic arthritis.


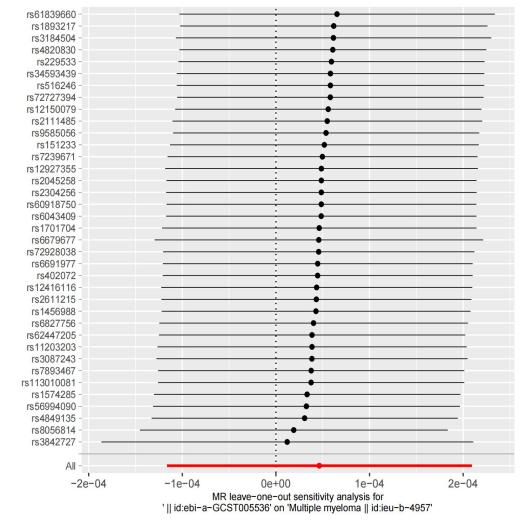

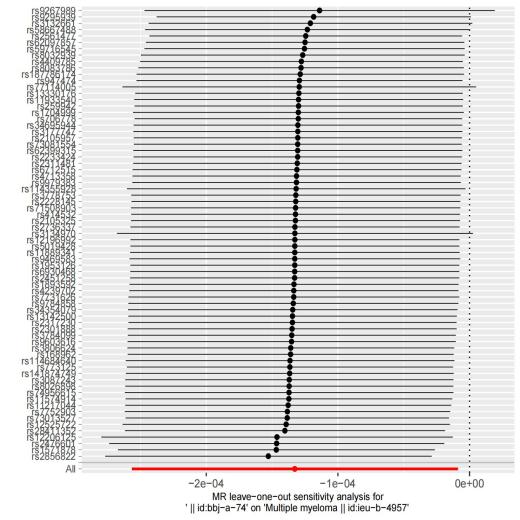


A B


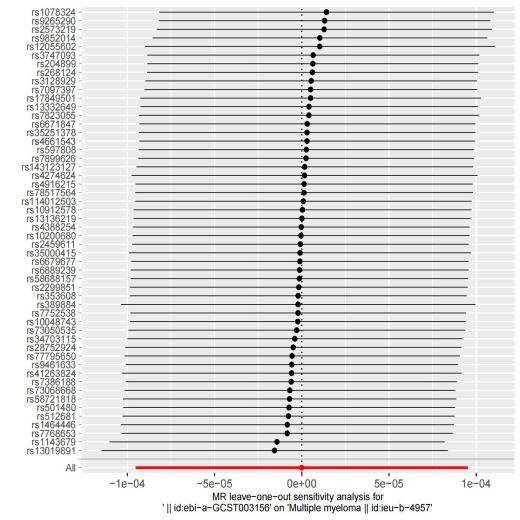

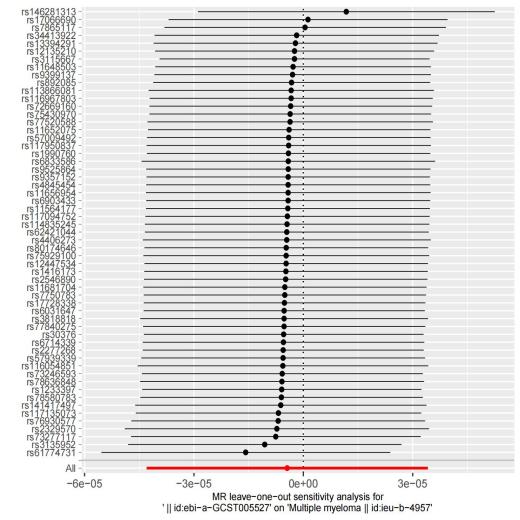


C D


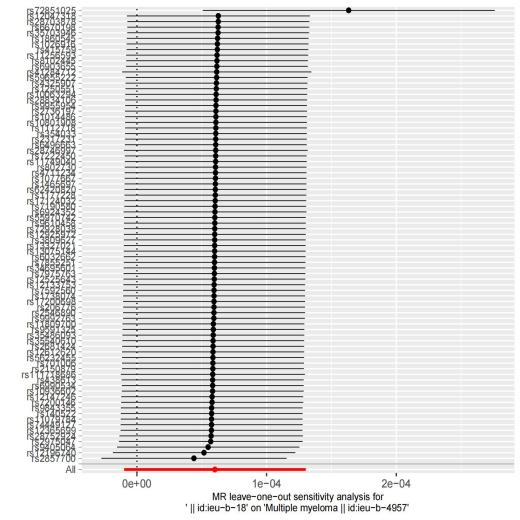

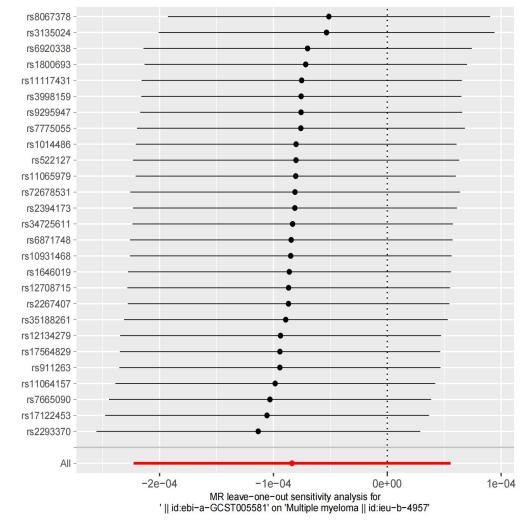


E F


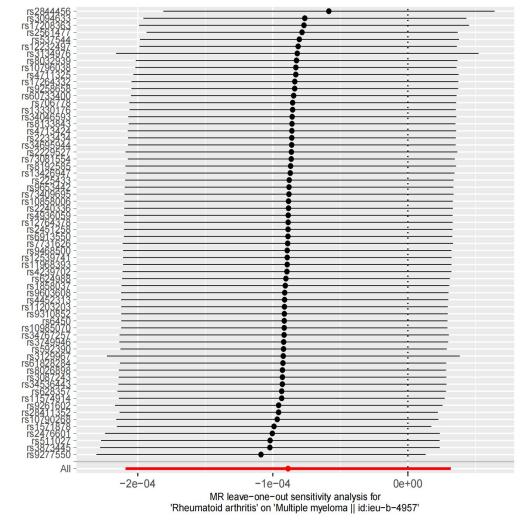


G

**Supplementary figure3**. Forest plot of the causal effect on MM outcomes after excluding instrument SNPs for eight autoimmune diseases on a case-by-case basis. (A)Type 1 diabetes mellitus;(B)Rheumatoid arthritis;(C)Systemic lupus erythematosus ;(D)Psoriasis;(E)Multiple sclerosis ;(F)Primary biliary cirrhosis;(G)Juvenile idiopathic arthritis.
